# Sensitive Montmorillonite Evaporation Detector Based on Montmorillonite Monolayer Nanosheets

**DOI:** 10.3390/polym18030383

**Published:** 2026-01-31

**Authors:** Jiahao Zhao, Qinglin Jia, Xu Wang, Jinhui Zhang, Yizhen Xu, Hai Zhao, Benbo Zhao, Shixiong Sun, Minghao Zhang, Min Xia, Zhengmao Ding, Chao Wang

**Affiliations:** 1 School of Materials Science and Engineering, North University of China, Taiyuan 030051, China; sz202303113@st.nuc.edu.cn; 2 Pen-Tung Sah Institute of Micro-Nano Science and Technology, Xiamen University, Xiamen 361005, China; jiaqinglin@stu.xmu.edu.cn (Q.J.); 2421011033@stu.xmut.edu.cn (X.W.); 33520251153322@stu.xmu.edu.cn (J.Z.); 34520232201194@xmu.edu.cn (Y.X.);; 3 School of Mechanical and Automotive Engineering, Xiamen University of Technology, Xiamen 361024, China; 4 School of Chemistry and Chemical Engineering, North University of China, Taiyuan 030051, China; 5 School of Materials Science and Engineering, Beijing Institute of Technology, Beijing 100081, Chinaxminbit@bit.edu.cn (M.X.)

**Keywords:** ion transport, montmorillonite, monolayer nanosheets, energy coating, evaporation

## Abstract

Two-dimensional (2D) materials open up exciting possibilities for the study of ion transport behavior for green energy. Here, a simple and effective strategy to fabricate high-conductivity nanofluidic channels based on exfoliated montmorillonite (MTM) nanosheets is proposed. The resource-rich and low-cost layered MTM was first exfoliated into monolayer nanosheets using Exolit OP 550. Subsequently, the MTM nanosheets with Exolit OP 550 were assembled into 2D nanofluidic devices by the layer-by-layer self-assembly method. The results show that Exolit OP 550 exfoliates different types of layered MTM into monolayer nanosheets with uniform contrast and integrity. The reconstructed Na-MTM nanofluidic device has the highest ionic conductance. The ionic conductivity of the Na-MTM 2D nanofluidic device was effectively improved after Li^+^ modification with a higher charge density. After further optimizing the content of Exolit OP 550, the ion conductivity of the MTM nanofluidic device reached 4.66 × 10^−4^ S cm^−1^, which is 55.3% higher than the highest known value among the same nanofluidic devices. Interestingly, this nanofluidic device exhibited a very high sensitivity in detecting water evaporation, which can reach 10^−12^ S s^−1^ in resolution. This economically viable strategy may advance the study of low-dimensional ion transport properties in new energy coatings and the design of evaporation detectors.

## 1. Introduction

With the proposal of “Nanofluids coming of age” by Bocqet in 2020, nanofluidics has received increasing attention [1]. Nanofluidics explores the transport of fluid and ionic species at the nanoscale, which could have disruptive impacts on key societal issues in water remediation and energy [2]. In recent years, with the emergence of a large number of new nanomaterials and fine processing technologies, nanofluidics has entered a booming phase. Devices suitable for the systematic study of nanofluids are the biggest challenge hindering the development of the field. After 17 years of development, 2D nanofluidic devices based on two-dimensional (2D) nanosheets such as graphene (GO), MXenes, carbon nitride, black scale, molybdenum disulfide, and clay prepared with high throughput, high integration density, and good scalability are gradually becoming mainstream within the field of nanofluidics [3,4,5,6,7,8,9,10,11,12,13]. However, 2D nanosheets still face the challenge of being difficult to prepare at low cost and large scale [14], which hinders the transition of 2D nanofluidic devices to the application stage [15].

As an abundant silicate material, montmorillonite (MTM) has a low cost [16,17]. Importantly, montmorillonite nanosheets have high charge-loading properties and are excellent candidates for the preparation of nanofluidic devices [18]. Therefore, MTM has attracted more attention in the field of nanofluidics. For example, Liu et al. vacuum-filtered MTM nanosheets into a 6.5 mm × 6.0 mm ion exchange membrane through layer-by-layer self-assembly [7]. After mounting the membrane parallel to the direction of the current flow, a conductivity of 3.0 × 10^−4^ S cm^−1^ was obtained in a 10^−7^ M KCl solution. The transport characteristics of the ion exchange membrane when it is perpendicular to the direction of current flow were also studied. Xiao et al. obtained a conductance of ~2.5 × 10^−6^ S (10^−6^ M KCl solution) in a vertically placed Na-MTM ion exchange membrane of 0.03 mm^2^ [19]. Qin et al. used aramid nanofibers (ANFs) to modify MTM nanosheets and prepared an organic–inorganic hybrid membrane with a conductivity of ~1.0 × 10^−6^ S when vertically placed in 10^−6^ M KCl solution [20]. Hao et al. modified MTM nanosheets with negatively charged polyacrylic acid (PAA) and positively charged polyethylenimine (PEI), respectively, and prepared a heterogeneous membrane with ion rectification properties [21]. This membrane achieved a conductance of ~4.0 × 10^−7^ S in 10^−6^ M KCl solution with a test area of 3.14 × 10^4^ µm^2^. Xiao et al. used spiropyran-modified surfactants to compound Na-MTM nanosheets, and prepared an ion-exchange membrane with a conductivity of ~1.0 × 10^−7^ S in 10^−6^ M KCl solution with a test area of 0.03 mm^2^ [22]. Wu et al. modified MTM membrane with PAA and dioctadecyldimethylammonium bromide (DODAB), and achieved a conductance of ~1.3 × 10^−6^ S in 10^−4^ M KCl solution with a test area of 0.785 mm^2^ [23]. The exfoliation method of MTM in these reports is all ultrasonic, which not only cannot be prepared on a large scale but also causes defects in the crystal structure of the nanosheets. Importantly, their electrical conductivity is not high. Therefore, the construction of nanofluidic devices based on low-cost, large-scale preparation of nanosheets and further research on the transport of fluids and ionic substances inside them are of great scientific significance and potential application value. In previous studies, we innovatively developed a new method for the large-scale exfoliation of resourceful and inexpensive MTM materials into defect-free monolayer nanosheets in a highly efficient and productive form [24,25]. This method has the potential for large-scale industrialized production without technical barriers.

Herein, three kinds of layered MTM were firstly exfoliated into monolayer nanosheets using polyethylene glycol phosphate (Exolit OP 550), and then MTM/Exolit OP 550-based 2D nanofluidic devices were prepared by cyclic washing and layer-by-layer self-assembly methods. The influences of the types of MTM, cation modifications, and the content of Exolit OP 55 on the structure and ion transport characteristics were investigated. This can be useful for the large-scale preparation of low-cost 2D nanofluidic devices, and it is expected to speed up the process of the industrialization and application of the 2D nanofluidic devices in the field of new energy coatings. In addition, the application potential of this nanofluidic device in detecting evaporation is also explored.

## 2. Experimental Section

### 2.1. Materials

Organic montmorillonite (OMTM, past 200 mesh), lithium-based montmorillonite (Li-MTM, past 200 mesh), and sodium-based montmorillonite (Na-MTM, past 200 mesh) were all industrial-grade and supplied by Zhejiang Fenghong New Material Co., Ltd. (Huzhou, China). Polyethylene phosphoglycolide (Exolit OP 550, $\bar{M_{n}}$ = 831) was industrial-grade and obtained from Clariant. Polydimethylsiloxane elastomer (PDMS, DC184) was industrial-grade and provided by Dow Corning. Potassium chloride (KCl), lithium chloride (LiCl), sodium chloride (NaCl), and anhydrous ethanol were all analytical reagents and purchased from Aladdin. Deionized water (18.2 MΩ cm) was homemade in the laboratory.

### 2.2. Large-Scale Exfoliation of MTM in Exolit OP 550

A quantitative amount of Exolit OP 550 and MTM after vacuum dewatering was added to a beaker and manually stirred with a glass rod for about 3 min until becoming homogeneous.

### 2.3. Fabrication of the MTM Nanofluidic Devices

**Figure 1:**
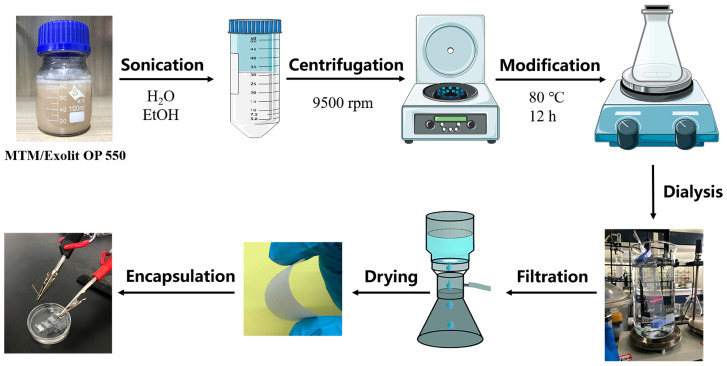
Schematic diagram of the fabrication process of MTM nanofluidic devices.

Different kinds of MTM/Exolit OP 550 composites were sonicated in deionized water for 10 min and centrifuged at 9500 rpm for 10 min to reduce the Exolit OP 550 content. The sonication and centrifugation processes were repeated several times to obtain suspensions with different numbers of washes. The obtained suspensions were mixed with 1 M KCl, LiCl, or NaCl solutions and stirred for 24 h at 80 °C. Then, the different kinds of suspensions were dialyzed with deionized water until the presence of Cl^-^ was not detected. Subsequently, the different cation-modified MTM suspensions were vacuum-filtered on a hybrid fiber microporous filter membrane. After drying at room temperature for 1 day, the different cation-modified MTM membranes were obtained. The dried MTM membranes were cut into rectangular strips of 8 mm × 4.5 mm and encapsulated in PDMS elastomer at 100 °C. Two reservoirs were cut on both sides of the rectangular MTM membranes to obtain MTM nanofluidic devices. The ion transport properties were characterized by adding different concentrations of KCl solutions. The preparation is shown in Figure 1.

### 2.4. Characterizations

#### 2.4.1. Small-Angle X-Ray Diffraction (Small-XRD) Testing

The MTM/Exolit OP 550 composite was flat-coated in a glass bath and placed into an X-ray diffractometer and tested in the 2θ range of 0.6–10°. The test temperature was 25 °C. The electrode material was Cu kα. The tube pressure was 40 kV. The tube current was 40 mA. And the scan rate was about 1 °/min.

#### 2.4.2. Transmission Electron Microscopy (TEM) Testing

A total of 0.02 g of MTM/Exolit OP 550 composite was dissolved in 5 mL of deionized water or ethanol solution, homogeneously dispersed, and spot-sampled on an ultrathin carbon-supported membrane copper mesh and tested on a transmission electron microscope. The line resolution was 0.14 nm.

#### 2.4.3. Scanning Electron Microscope (SEM) Testing

The MTM powder and membranes were imaged by an SU8020 SEM (Hitachi, Ltd., Hitachi, Tokyo, Japan) at 3.0 kV and 5.0 kV, respectively. MTM membranes were brittle in liquid nitrogen, and the fracture morphology of the membrane material was tested in a scanning electron microscope after sputtering with Pt on the cross-section. The thickness of Pt was about 5 nm. An electron gun was used as the cold field-emitting electron source with a magnification of 20,000 times.

#### 2.4.4. Atomic Force Microscopy (AFM) Testing

AFM imaging of MTM nanosheets was performed using a Cypher ES microscope (Asylum Research, Santa Barbara, CA, USA) in tapping mode under ambient conditions. Samples were prepared by drop-casting approximately 10 μL of an ethanol dispersion onto a mica substrate (Beijing Zhongjing Keyi Technology Co. Ltd., Beijing, China).

#### 2.4.5. Selected Area Electron Diffraction (SAED) Testing

SAED patterns were acquired using an FEI Tecnai F30 transmission electron microscope (FEI, Hillsboro, USA) operated at 200 kV. Sample preparation involved depositing ~5 μL of an ethanol dispersion of MTM nanosheets onto ultrathin carbon-coated copper grids (200 mesh; Beijing Zhongjing Keyi Technology Co. Ltd., China), with pre-blotting on filter paper to rapidly remove excess solvent within 1 s.

#### 2.4.6. Dynamic Light Scattering (DLS) Testing

The Zeta potential of the nanosheets was measured using a Zeta-sizer Nano ZS90 laser nanoparticle analyzer (Malvern, Great Malvern, UK). Before testing, the samples were diluted with ethanol to a concentration of approximately 0.02 wt.%, and all measurements were conducted at 25 °C using a DTS1070 cell. Each sample was tested in triplicate, with the average value reported as the final result.

#### 2.4.7. Thermo-Gravimetric Analysis (TGA)

TGA was conducted using a TGA/DSC 1 thermal analyzer (Mettler Toledo, Greifensee, Switzerland). The temperature was raised from 40 °C to 600 °C at a heating rate of 10 °C/min under a nitrogen atmosphere.

#### 2.4.8. Surface Charge Density (σ) Testing

The σ values of KCl-, NaCl-, and LiCl-modified Na-MTM membranes were recorded using a SurPASS 3 electrokinetic analyzer (Anton Paar, Graz, Austria). The concentration of KCl used for testing is set to 0.001 M. The σ of the membrane is estimated based on the Grahame equation.

#### 2.4.9. Conductivity Testing

Linear sweep voltammetry (LSV) measurements were performed on the MTM nanofluidic device using a CHI electrochemical workstation with Ag/AgCl reference electrodes positioned in both reservoirs. Current–voltage curves were recorded over −0.5~0.5 V at a scan rate of 0.01 V s^−1^ in a pH 7.0 KCl electrolyte. Prior to testing, the MTM membrane was pre-equilibrated in deionized water for 12 h to ensure complete hydration. All electrolysis cells were sealed with airtight covers to prevent atmospheric CO_2_ dissolution and minimize solvent evaporation during measurements conducted at 25 ± 1 °C.

## 3. Results and Discussion

### 3.1. Large-Scale Exfoliation of MTM

**Figure 2:**
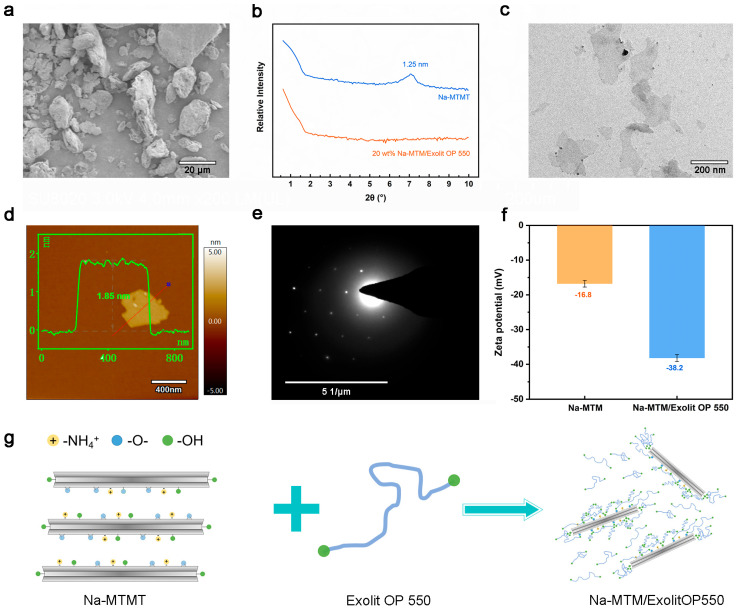
(**a**) SEM images of Na-MTM powder. (**b**) Small-angle XRD patterns. (**c**) TEM images, (**d**) AFM images, and (**e**) SAED pattern of Na-MTM nanosheets. (**f**) Dispersion Zeta potentials of Na-MTM nanosheet dispersions. (**g**) Schematic diagram of Exolit OP 550 stripping Na-MTM.

Figure 2a presents the microstructure of Na-MTM powder, revealing irregular agglomerates composed of primary particles (~1.5 μm lateral size, ~100 nm height). These hierarchical structures form through cohesive assembly of nanoscale lamellae driven by van der Waals and electrostatic forces. Ultrasonic treatment at 240 W for 2 h did not achieve exfoliation of the Na-MTM (Figure S1). The small-angle XRD curves and TEM image of 20 wt.% Na-MTM/Exolit OP 550 composite are shown in Figure 2b. Na-MTM has a diffraction peak of the (001) crystal plane at 7.10°. According to Bragg’s equation [26], this diffraction peak corresponds to a layer spacing of 1.25 nm. The (001) diffraction peak of Na-MTM powder completely disappeared after about 3 min of manual stirring with a glass rod in Exolit OP 550. This result indicates that the layered Na-MTM was exfoliated into monolayer nanosheets. According to our previous studies [16], this is related to the exfoliation force generated by the aggregation of Exolit OP 550 in the MTM interlayer. In Figure 2c, the exfoliated Na-MTM nanosheets have a more uniform lining, irregular shape, and better integrity, and their size is about 100–500 nm. Notably, this strategy can exfoliate 20 wt.% of MTM within 3 min with gentle stirring at room temperature. The peeling concentration is higher than others. Meanwhile, the stripping time is the highest, which is 97.5% shorter than others [27,28,29,30,31,32,33,34,35,36,37]. As evidenced by AFM in Figure 2d and Figure S2, Na-MTM nanosheets demonstrate exceptional planarity with a measured thickness of 1.85 ± 0.15 nm. This value exceeds the theoretical monolayer thickness (0.96 nm) but falls below the characteristic bilayer threshold (≥1.92 nm), indicating predominantly monolayer configurations with minimal deviation from ideal crystallographic dimensions. Figure 2e displays a typical SAED pattern of Na-MTM ultrathin nanosheets. The pattern exhibits a regular hexagonal symmetry with well-defined diffraction spots, showing monotonically decreasing intensity from the center to the periphery. This diffraction profile matches the theoretically derived pattern from the molecular model of a monolayer MTM, confirming that the Na-MTM ultrathin nanosheets obtained via Exolit OP 550 exfoliation are single-layered. Furthermore, the nanosheets retain excellent crystallinity and are virtually free of discernible structural defects. Analysis of dynamic light scattering (DLS) results in Figure 2f and Table S1 reveals that the Zeta potential of modified MTM undergoes a significant reduction from −16.8 ± 0.5 mV to −38.2 ± 0.5 mV. This phenomenon occurs because Exolit OP 550 causes the Na-MTM interlayer to exfoliate into monolayer nanosheets, resulting in a significant increase in specific surface area. Concurrently, polar functional groups of Exolit OP 550 anchor onto nanosheet surfaces via electrostatic/hydrogen bonding interactions, thereby enhancing surface negative charge density by 128% compared to pristine samples. In addition, Exolit OP 550 reduces the polydispersity index (PDI) from 0.582 to 0.183, suggesting a more uniform and stable state. This may be attributed to Exolit OP 550, which transforms Na-MTM particles into monolayer nanosheets and increases their charge. The enhanced charge boosts electrostatic repulsion, preventing the nanosheets from aggregating. As shown in Figure 2g, layered MTM and Exolit OP 550 (with optional ethanol additive) were manually homogenized via stirring using a glass rod at 25 °C for ∼3 min. The mixture underwent isothermal processing in sealed glass vessels at 25 °C for 0–12 h.

### 3.2. Structural Analysis of MTM Nanofluidic Devices

**Figure 3:**
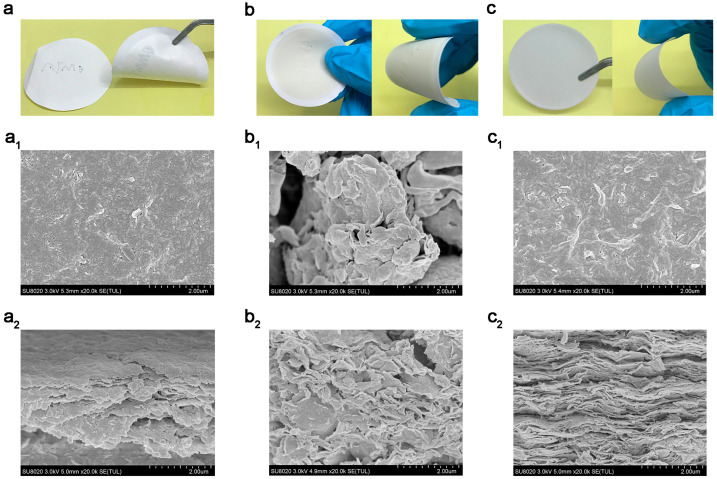
Photographs of (**a**) OMTM membrane, (**b**) Li-MTM membrane, and (**c**) Na-MTM membrane. Surface topography of (**a_1_**) OMTM membrane, (**b_1_**) Li-MTM membrane, and (**c_1_**) Na-MTM membrane. Cross-sectional morphologies of (**a_2_**) OMTM membrane, (**b_2_**) Li-MTM membrane, and (**c_2_**) Na-MTM membrane. The nanosheets used were washed by centrifugation 8 times. The thicknesses are approximately 5.0, 5.5, and 7.0 μm, respectively.

Figure 3 shows the appearance, surface, and cross-section phase diagrams of different MTM membranes prepared using vacuum self-assembly. All three MTM membranes appear to be white in color and flexible. The surface of the OMTM membrane is smoother, which is related to its internal organic surfactant. The surface of the Li-MTM membrane is rougher, which is related to its poor exfoliation and obvious agglomeration. The surface of the Na-MTM membrane is moderately smooth. From the cross-section topography, it can be seen that the Li-MTM nanofluidic channels are arranged haphazardly, while the OMTM and Na-MTM nanosheets are stacked in an orderly manner, and the resulting nanofluidic channels are more regular with fewer defects, with more stable nanofluidic channel dimensions, ensuring the symmetry of the nanofluidic devices, and are more suitable for the study of the nanoscale ion transport properties [38]. In addition, membrane thickness is affected by filtration time and can lead to a slight increase in channel height (Table S2). This may be related to the fact that thicker membranes weaken a certain amount of filtration force.

### 3.3. Ion Transport in MTM Nanofluidic Devices

**Figure 4:**
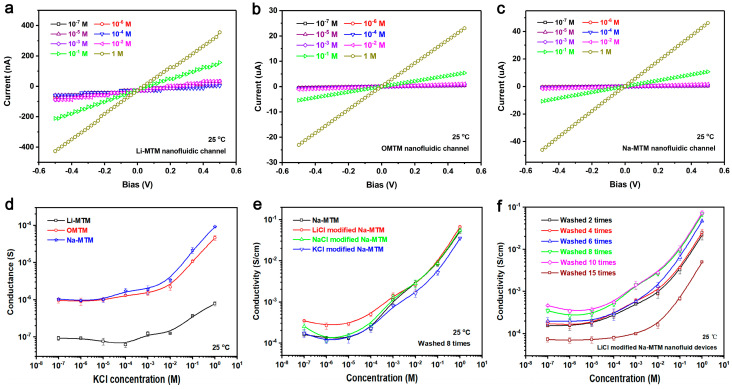
Current–voltage curves of (**a**) Li-MTM, (**b**) OMTM, and (**c**) Na-MTM nanofluidic devices in different concentrations of KCl. (**d**) Ionic conductance comparison of different MTM nanofluidic devices. The test temperature was 25 °C. The nanosheets used were washed by centrifugation 8 times. (**e**) Variation in ionic conductivity of Na-MTM nanofluidic devices modified with different cations as a function of KCl solution concentration. The test temperature was 25 °C. The nanosheets used were washed by centrifugation 8 times. (**f**) Ionic conductivity of LiCl-modified Na-MTM nanofluidic devices with different contents of Exolit OP 550 as a function of KCl solution concentration. The test temperature was 25 °C.

MTM species have a greater impact on ion transport in nanofluidic channels. Figure 4 shows the current–voltage curves of Li-MTM, OMTM, and Na-MTM nanofluidic devices in different concentrations of KCl and the comparison of ionic conductance. As can be seen, the current–voltage curves of OMTM, Li-MTM, and Na-MTM nanofluidic devices show a linear ohmic behavior with negligible ionic current rectification, indicating that the prepared nanofluidic devices have a symmetric geometry [5,9,10,20]. The ionic conductance of the nanofluidic device can be obtained from the current–voltage curve. As seen in Figure 4d, there is a turning point in the conductance curve of the MTM nanofluidic device. When the concentration of the KCl solution is greater than 10^−4^ M, the conductance of the MTM nanofluidic device increases gradually with the increase in the concentration, which shows an obvious phenomenon of concentration-controlled conductance. When the concentration of the KCl solution is less than 10^−4^ M, the ionic conductance of the MTM nanofluidic device is close and the curve exhibits a plateau region. This is because the Debye length of the fluid ions increases as the solution concentration decreases, and when the Debye length of the fluid ions is similar to the size of the prepared nanofluidic channel, the motion of the fluid ions is then affected by the surface charge of the channel, exhibiting the typical behavior of the surface charge controlling the motion of the ions [3]. Because the channel surface charge is certain, the ionic conductance does not show a significant change when the concentration continues to decrease, but instead remains at an approximately constant value.

In addition, the conductance of Na-MTM and OMTM nanofluidic devices is an order of magnitude higher than that of Li-MTM nanofluidic devices at different concentrations of KCl solution. This is because the nanofluidic channels obtained by vacuum self-assembly of Li-MTM do not exhibit a complete laminar structure, appearing as agglomerates of nanosheets, do not form ion transport pathways, and ultimately impede the transport of ions, which is reflected in the very low ionic conductance. In contrast, the Na-MTM nanofluidic channel has a better stacked laminar structure, a more stable channel size, a low resistance to ion transport, and a high selectivity for ions, and thus exhibits the highest ionic conductance. The surfactant on the surface of OMTM has a hindering effect on the ion transport within the channel, resulting in a smaller ionic conductance than that of the Na-MTM nanofluidic channel.

Natural Na-MTM nanosheets have a large number of negative charges on the surface due to the isomorphous substitution effect. In order to maintain charge balance, Na^+^ is adsorbed on the surface. Modification of the surface charge density of MTM nanosheets can be achieved through cationic Na^+^ replacement on different scales. Therefore, the effect of different-sized cations on the conductance of Na-MTM nanofluidic devices was next investigated. After optimizing the temperature and time (Table S3), the optimal conditions for the ion exchange reaction are determined to be 80 °C and 24 h. Figure 4e shows the ionic conductivity of Na-MTM nanofluidic devices modified with different cations as a function of KCl solution concentration. The Na-MTM nanofluidic devices modified by different cations still exhibit the typical behavior of surface charge-controlled ion transport. The Na-MTM nanofluidic channel modified by LiCl exhibits the highest ionic conductivity at low concentrations, whereas the difference in ionic conductivity between NaCl-modified, KCl-modified, and unmodified Na-MTM at low concentrations is not significant, which is consistent with the trend of surface charge density changes in the modified Na-MTM nanofluid channels (Table S4) [39,40]. The sodium MTM nanofluid channel modified with lithium chloride exhibits the highest ion conductivity at low concentrations, while the difference in ion conductivity between sodium chloride-modified, potassium chloride-modified, and unmodified sodium MTM at low concentrations is not significant, which is consistent with the trend of surface charge density changes in the modified Na MTM nanofluid channel (Table S4).

This is because Li^+^ has a small radius and high adsorption energy, which can have a displacement reaction with Na^+^ in the interlayer of Na-MTM; at the same time, Li^+^ with a smaller radius has a higher charge density and a strong hydration ability, which is conducive to stabilizing the water molecules in the interlayer, which makes the LiCl-modified Na-MTM nanofluidic devices have a more stable and regular channel size and a higher surface charge density [7]. For NaCl-modified Na-MTM nanofluidic devices, the conductivity is close to that of unmodified Na-MTM nanofluidic devices, indicating that the charge on the surface of Na-MTM nanosheets is approximately saturated. For the KCl-modified Na-MTM nanofluidic devices, due to the larger radius of K^+^ than Na^+^, the adsorption energy is low, and it cannot displace the interlayer Na^+^, resulting in the conductivity in the low-concentration region being close to that of the unmodified Na-MTM nanofluidic devices.

Na-MTM was stripped and prepared into nanofluidic devices together with the stripping reagent Exolit OP 550 by layer-by-layer self-assembly. To investigate the effect of Exolit OP 550 content on the ion transport properties in the devices, the Na-MTM/Exolit OP 550 composite was subjected to several cycles of washing treatment as a way to control the content of Exolit OP 550 in the devices. TGA results (Table S5) indicate that water washing successfully reduces the levels of Exolit OP 550, which aligns with the initial hypothesis. Typically, after 10 washing cycles, the residual content of Exolit OP 550 on Na-MTM nanosheets is approximately 5.28 wt.%. Furthermore, as the content of Exolit OP 550 decreases, both the negative Zeta potential and the PDI of the Na-MTM/Exolit OP 550 aqueous dispersion gradually increase simultaneously. This phenomenon occurs because the reduction in the weakly acidic Exolit OP 550 content allows more OH groups to undergo deprotonation, thereby enhancing the surface charge. Concurrently, the decrease in Exolit OP 550 content also raises the propensity for aggregation of Na-MTM nanosheets. Figure 4f shows the variation in ionic conductivity of Na-MTM nanofluidic devices with KCl solution concentration at different organic content. The nanofluidic devices at different Exolit OP 550 contents have the typical behavior of surface charge-controlled ionic transport. The ionic conductivity of LiCl-modified Na-MTM nanofluidic devices showed a tendency to increase and then decrease with the number of washes. The LiCl-modified Na-MTM nanofluidic device after 10 washes has the largest ionic conductivity, which is 4.66 × 10^−4^ in 10^−7^ M KCl solution, which is about 1.55 times the highest value reported in the literature [7]. This is because the acidic Exolit OP 550 weakens the charge density on the surface of the MTM nanofluidic channel, leading to a decrease in the selectivity of the channel for ions, which is presented as a decrease in conductivity. And when the number of washes is too high, the homogeneity of the MTM nanosheets deteriorates, and the regularity of the prepared channels deteriorates, leading to an increase in the resistance to ion transport in the channels, presenting as a decrease in conductivity.

### 3.4. Evaporation Detection Application

**Figure 5:**
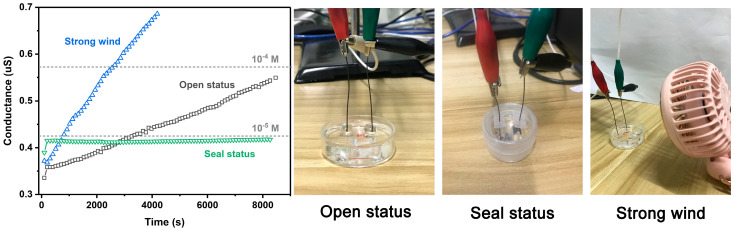
Ionic conductivity of Na-MTM nanofluidic devices in different scenarios as a function of test time. The initial liquid was a 10^−6^ M KCl aqueous solution. The temperature and humidity of the experimental environment were 25 °C and 25% RH, respectively. The wind speed of the additional wind was set as 1.7 m s^−1^.

During the research process, it was surprisingly discovered that the prepared Na-MTM nanofluidic device was extremely sensitive to the concentration of the salt solution, which inspired us to use it as an evaporation detector (Figure 5). In the laboratory environment (25 °C, 25% RH), a 10^−6^ M KCl solution was selected as the detection object. With the free and slow evaporation of water, the conductance in the open state shows a slowly rising trend, and reaches the same conductance as 10^−5^ M KCl at 3264 s. The calculated evaporation rate can be expressed as 0.022 nS s^−1^. In contrast, the conductance in the almost completely sealed state hardly changes with the test time. For comparison, a strong wind environment with a velocity of 1.7 m s^−1^ was applied outside the electrolytic cell to speed up the volatilization of water. As expected, the conductance in a strong wind environment increases rapidly, reaching the same conductance as 10^−5^ M and 10^−4^ M KCl at 780 s and 2550 s, respectively. The evaporation rate has been increased by 291% to 0.086 nS s^−1^. This is attributed to the increase in ion concentration resulting from water evaporation. Furthermore, we utilized this device to characterize the variation in free, slow evaporation-induced conductivity caused by humidity and ion species (Figure S3). As expected, higher humidity leads to lower evaporation-induced conductivity. Specifically, the conductivities of KCl, NaCl, and LiCl decrease progressively. This occurs because smaller cation volumes correspond to larger specific surface areas, leading to larger hydrated ion sizes and slower evaporation rates. Importantly, there are no reported two-dimensional nanofluidic channels that can be used to detect water evaporation. This interesting experimental result can provide a reference for the design of highly sensitive evaporation detection devices and is expected to be used in the detection of complex environmental parameters (such as temperature, humidity, wind speed, etc.).

## 4. Conclusions

In summary, MTM monolayer nanosheets were prepared on a large scale and at a low cost based on the self-aggregation of Exolit OP 550, and low-cost nanofluidic devices were later prepared by layer-by-layer self-assembly of Exolit OP 550 and its exfoliated MTM monolayer nanosheets.

- The effects of MTM species, cationic modification, and Exolit OP 55 content on the structure of monolayer nanosheets and ion transport properties in nanofluidic devices were investigated, respectively.
- After stirring for 3 min at room temperature, Exolit OP 550 can exfoliate MTM into monolayer nanosheets with uniform lining and good integrity.
- The Na-MTM nanofluidic channels prepared by layer-by-layer self-assembly have a regular microstructure, leading to a higher ionic conductance than that of Li-MTM and OMTM nanofluidic devices. Moreover, modification of Na-MTM nanofluidic devices using a higher charge density Li+ can effectively increase their electrical conductivity. In addition, by modulating the content of Exolit OP 550, the conductivity can reach 4.66 × 10^−4^ S cm^−1^, which is 1.55 times the highest known value.
- In addition, this nanofluidic device sensitively detected the evaporation of water with a resolution of 10^−12^ S s^−1^ at 25 °C.

Economically viable strategies are expected to potentially promote the study of low-dimensional ion transport properties and the design of evaporation detectors.

## Data Availability

Data are contained within the article (and Supplementary Materials). Further inquiries can be directed to the corresponding author.

## Supplementary Materials

The following supporting information can be downloaded at: https://www.mdpi.com/article/10.3390/polym18030383/s1, Figure S1: Small-angle XRD pattern and TEM image of ultrasound-treated Na-MTM; Figure S2: Histogram of the Na-MTM nanosheets thicknesses; Figure S3: Ionic conductivity of Na-MTM nanofluidic devices in different (a) humidity and (b) ion species; Table S1: Zeta potentials of Na-MTM nanosheets before and after Exolit OP 550 modification; Table S2: The thickness and channel height of OMTM, Li-MTM, and Na-MTM membranes at different filtration times; Table S3: The negative Zeta potential of nanosheets after ion exchange at different temperatures and times; Table S4: σ of the Na-MTM membranes; Table S5: pH, Zeta potential, and Exolit OP 550 content of the Na-MTM/Exolit OP 550 composite material after different washings.
