# Supplementary material for: Sensitive Montmorillonite Evaporation Detector Based on Montmorillonite Monolayer Nanosheets
Source: Polymers (Basel). 2026 Jan 31;18(3):383. doi: 10.3390/polym18030383 (PMC12899846; doi:10.3390/polym18030383)
Supplement: Supplementary file 1 [file polymers-18-00383-s001.zip › polymers-4121287-supplementary.pdf]

Supplementary Materials

Sensitive montmorillonite evaporation detector based on montmorillonite monolayer nanosheets

Jiahao Zhao [1, 2], Qinglin Jia [2], Xu Wang [2,3], Jinhui Zhang [2], Yizhen Xu [2], Hai Zhao [2,4], Benbo Zhao [4], Shixiong Sun [4], Minhao Zhang [5], Min Xia [5], Zhengmao Ding [2*], Chao Wang [1*]

[1] *School of Materials Science and Engineering, North University of China, Taiyuan, 030051, PR China.*

[2] *Pen-Tung Sah Institute of Micro-Nano Science and Technology, Xiamen University, Xiamen, 361005, PR China.*

[3] *School of Mechanical and Automotive Engineering, Xiamen University of Technology, Xiamen 361024, PR China*

[4] *School of Chemistry and Chemical Engineering, North University of China, Taiyuan, 030051, PR China.*

[5] *School of Materials Science and Engineering, Beijing Institute of Technology, Beijing, 100081, PR China.*

** Correspondence and requests for materials should be addressed to Z. Ding (email: dzm@xmu.edu.cn) and C. Wang (email: wangchao_nuc@126.com)*

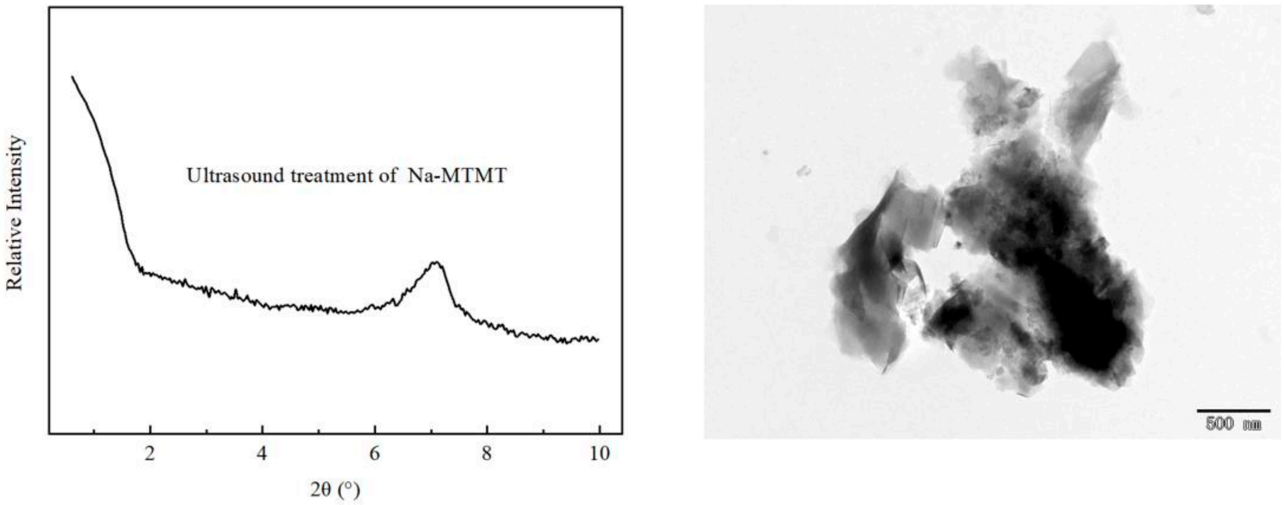

Figure S1. Small-angle XRD pattern and TEM image of ultrasound-treated Na-MTM. XRD results indicate that ultrasonic treatment fails to achieve complete exfoliation of this MTM. TEM images of the supernatant after ultrasonic centrifugation further confirm that the MTM remains in the multilayer structure. The yield of collected monolayer nanosheets is extremely low, nearly negligible.

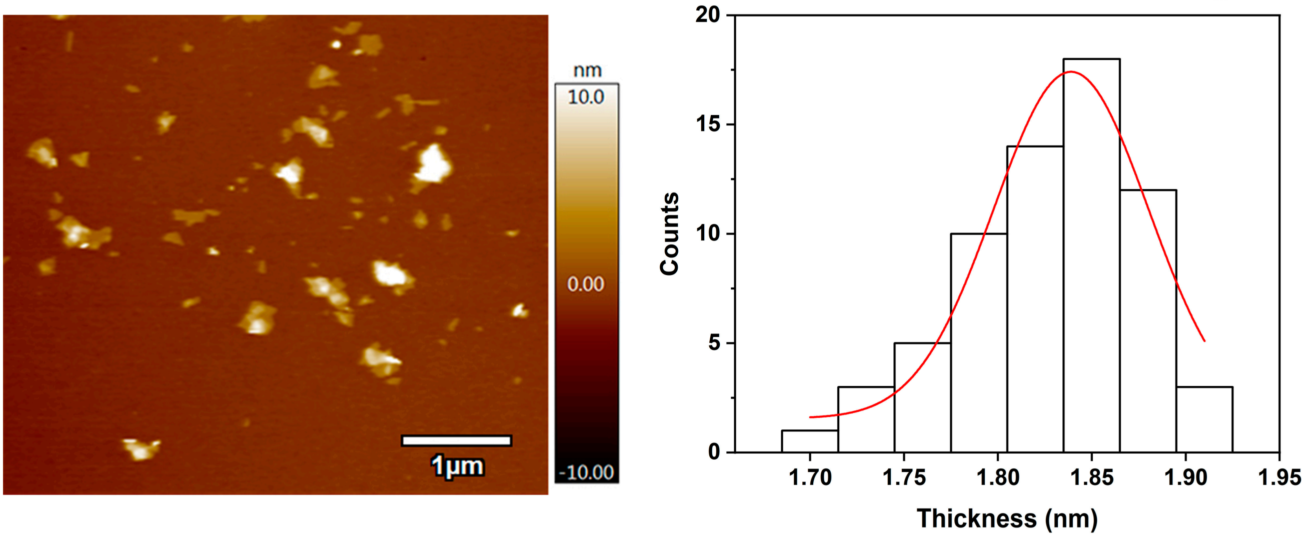

Figure S2. Histogram of the Na-MTM nanosheets thicknesses. The thicknesses of more than 60 Na-MTM nanosheets exhibit a normal distribution curve centered at 1.85 nm and ranging from 1.71 to 1.91 nm. The brighter areas in the figure are the nanoparticle stacks produced during the AFM sample preparation process. These nanoparticles are not included in the statistics.

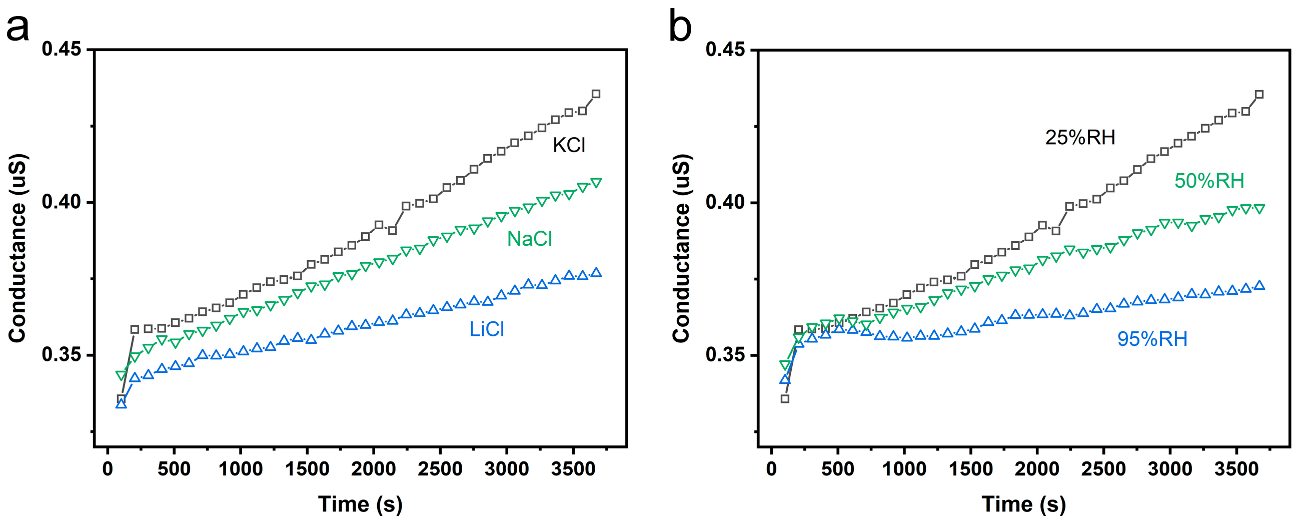

Figure S3. Ionic conductivity of Na-MTM nanofluidic devices in different (a) humidity and (b) ion species.

Table S1. Zeta potentials of Na-MTM nanosheets before and after Exolit OP 550 modification. The number of washes is 10.

	Zeta potential (mV)	PDI
Na-MTM	-16.8±0.16	0.582±0.12
Na-MTM/Exolit OP 550	-38.2±0.22	0.183±0.02

Table S2. The thickness and channel height of OMTM, Li-MTM, and Na-MTM membranes at different filtration times.

Membranes	Filtration time (min)	Thickness (μm)	Channel height (nm)
OMTM	15	~5.0	1.327±0.02
	30	~6.5	1.333±0.05
	45	~7.6	1.334±0.03
Li-MTM	15	~3.0	2.480±0.10
	30	~5.5	2.493±0.09
	45	~7.5	2.505±0.07
Na-MTM	15	~7.0	0.805±0.01
	30	~9.1	0.808±0.02
	45	~10.6	0.810±0.01

Table S3. The negative Zeta potential of nanosheets after ion exchange at different temperatures and times. The error is about 0.2 mV.

	KCl												LiCl												NaCl											
Temperature (°C)	25			50			80			110			25			50			80			110			25			50			80			110		
Time (h)	12	24	36	12	24	36	12	24	36	12	24	36	12	24	36	12	24	36	12	24	36	12	24	36	12	24	36	12	24	36	12	24	36	12	24	36
Zeta potential (mV)	37.7	36.1	35.7	36.8	35.7	33.7	35.6	34.2	33.5	33.9	32.6	31.4	39.8	42.1	45.1	40.0	43.9	46.8	43.6	47.1	47.0	46.8	46.1	44.3	38.1	38.2	38.4	38.1	38.3	38.6	38.5	38.7	38.7	38.6	37.2	37.0

Table S4. σ of the Na-MTM membranes.

Membrane	Na-MTM	KCl modified Na-MTM	NaCl modified Na-MTM	LiCl modified Na-MTM
σ (mC/m^2)	-2.62±0.02	-2.42±0.08	-2.68±0.03	-3.23±0.04

Table S5. pH, Zeta potential, and Exolit OP 550 content of the Na-MTM/Exolit OP 550 composite material after different washings.

Wash cycle	2	4	6	8	10	15
pH	3.84	4.32	4.89	5.11	5.28	5.33
Zeta potential (mV)	-22.2±0.23	-25.3±0.12	-28.5±0.15	-32.7±0.09	-38.2±0.22	-37.9±0.12
PDI	0.162±0.02	0.166±0.01	0.171±0.02	0.176±0.03	0.183±0.02	0.219±0.04
Content of Exolit OP 550 (wt.%)	13.65	10.28	8.75	6.67	4.96	2.46
